# Biological Roles Played by Sphingolipids in Dimorphic and Filamentous Fungi

**DOI:** 10.1128/mBio.00642-18

**Published:** 2018-05-15

**Authors:** Caroline Mota Fernandes, Gustavo H. Goldman, Maurizio Del Poeta

**Affiliations:** a Department of Molecular Genetics and Microbiology, Stony Brook University, Stony Brook, New York, USA; b Faculdade de Ciências Farmacêuticas de Ribeirão Prêto, Universidade de São Paulo, Ribeirão Prêto, São Paulo, Brazil; c Division of Infectious Diseases, School of Medicine, Stony Brook University, Stony Brook, New York, USA; d Veterans Affairs Medical Center, Northport, New York, USA; University of Texas Health Science Center at Houston

**Keywords:** ceramide, glucosylceramide, inositol phosphorylceramide, sphingolipids, dimorphic fungi, fatty acids, filamentous fungi, fungal infection, hyphal

## Abstract

Filamentous and dimorphic fungi cause invasive mycoses associated with high mortality rates. Among the fungal determinants involved in the establishment of infection, glycosphingolipids (GSLs) have gained increased interest in the last few decades. GSLs are ubiquitous membrane components that have been isolated from both filamentous and dimorphic species and play a crucial role in polarized growth as well as hypha-to-yeast transition. In fungi, two major classes of GSLs are found: neutral and acidic GSLs. Neutral GSLs comprise glucosylceramide and galactosylceramide, which utilize Δ4-Δ8-9-methyl-sphingadienine as a sphingoid base, linked to a C_16–18_ fatty acid chain, forming ceramide, and to a sugar residue, such as glucose or galactose. In contrast, acidic GSLs include glycosylinositol phosphorylceramides (GIPCs), composed of phytosphingosine attached to a long or very long fatty acid chain (C_18–26_) and to diverse and complex glycan groups via an inositol-phosphate linker. GIPCs are absent in mammalian cells, while fungal glucosylceramide and galactosylceramide are present but diverge structurally from their counterparts. Therefore, these compounds and their biosynthetic pathways represent potential targets for the development of selective therapeutic strategies. In this minireview, we discuss the enzymatic steps involved in the production of fungal GSLs, analyze their structure, and address the role of the currently characterized genes in the biology and pathogenesis of filamentous and dimorphic fungi.

## INVASIVE FUNGAL INFECTIONS

Invasive fungal infections represent a major threat to immunocompromised patients, leading to approximately one-and-a-half million deaths per year worldwide (1). Among invasive mycoses, those caused by dimorphic and filamentous fungi are associated with significant morbidity and high mortality rates. The dimorphic fungal species Blastomyces dermatitidis, Coccidioides immitis, Histoplasma capsulatum, Paracoccidioides brasiliensis, and Sporothrix schenckii are the most prevalent etiologic agents of blastomycosis, coccidioidomycosis, histoplasmosis, paracoccidioidomycosis, and sporotrichosis, respectively, and together cause more than 1 million new infections per year in the United States alone (2). Except for *Sporothrix*, the occurrence of these dimorphic fungal pathogens is geographically restricted, and it is estimated that they infect 10 million people worldwide, remaining latent and establishing systemic infections when the host becomes immunodeficient (3–6). Among filamentous fungal infections, invasive aspergillosis (usually caused by the mold Aspergillus fumigatus) is one of the four most life-threatening, with a mortality rate of up to 90% if the infection is not properly diagnosed and treated (1). Mucormycosis is another fungal infection of emerging medical importance that is most frequently caused by the filamentous fungus Rhizopus oryzae (7). Unlike other molds which affect only immunodeficient patients, R. oryzae can cause lethal infections in otherwise immunocompetent individuals (8), including military personnel who sustained combat-related injuries. In fact, approximately 6.8% of the U.S. soldiers wounded in Afghanistan between 2009 and 2011 developed trauma-associated fungal infections (9), indicating that individuals exposed to blast injuries are at risk for invasive mycoses.

The medical relevance of systemic infections caused by dimorphic and filamentous fungi has brought great interest in the mechanisms underlying host immune response and fungal pathogenesis. Dimorphic and filamentous mycelia usually grow in soil, at 22 to 25°C, and produce easily aerosolized conidia (or arthroconidia, in the case of Coccidioides immitis) (10, 11). The primary route for most fungal entry in the mammalian host is the respiratory tract, with the inhalation of airborne conidia. In contrast, R. oryzae and S. schenckii infections are usually established after the traumatic inoculation of soil material contaminated with these fungi. The host body temperature (37°C) induces filamentous fungi to germinate from conidia to germlings and dimorphic fungi to switch morphologically from the infectious propagules (asexual conidia) to the pathogenic yeast (or spherules, in the case of Coccidioides immitis) form. Alveolar macrophages are the first line of immune defense against fungal conidia in the lungs and, in healthy individuals, are able to kill conidia or inhibit fungal growth through phagocytosis (12, 13). Pathogen evasion of lung macrophages and other immune system cells, such as neutrophils and dendritic cells, can lead to fungal dissemination to other organs (12). The establishment of the fungal disease is a combination of the host immune status and/or virulence factors produced by the fungus. For example, neutropenic patients are highly susceptible to invasive aspergillosis, whereas T-cell immunodeficiency is the main condition predisposing individuals to infection by dimorphic fungi. Whereas the role of the host immune system in fungal recognition and growth inhibition and, thus, in controlling the infection has been extensively studied (12, 14), the pathways that contribute to fungal virulence, particularly those involved in the regulation of hypha-to-yeast transition and conidial germination in dimorphic and filamentous fungi, are less appreciated. In this review, we discuss the relevance of glycosphingolipids (GSLs) in fungal growth, dimorphism, and virulence, which are key to lung colonization and systemic dissemination in mammalian hosts.

## GSL STRUCTURE AND MEMBRANE LOCALIZATION

**FIG 1:**
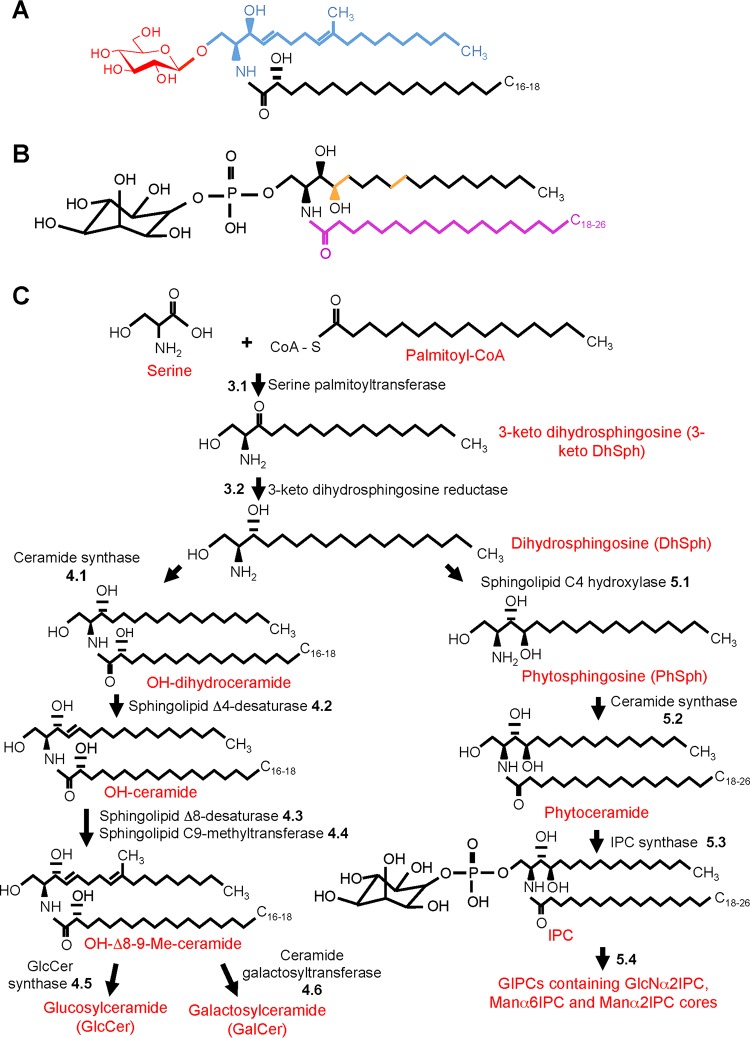
Glycosphingolipid structure and biosynthesis. (A) Basic structure of a neutral glycosphingolipid, made up of a sphingoid base (highlighted in blue) and a fatty acid chain (highlighted in black) to form ceramide, which is linked to a sugar residue (highlighted in red). (B) The structure of IPC, a simple acidic glycosphingolipid, is shown. Acidic glycosphingolipids differ from neutral glycosphingolipids in that they contain an additional -OH group at C_4_ of the sphingoid base and lack C_9_-methylation and Δ4- and Δ8-unsaturations (highlighted in orange). Another difference is that acidic glycosphingolipids are made up of a very long fatty acid (C_18–26_, highlighted in purple) instead of the C_16–18_ chain found in neutral glycosphingolipids. (C) Proposed biosynthetic pathway of glycosphingolipids. The reactions indicated by the number 3 are common to acidic and neutral GSL synthesis, while those indicated by the numbers 4 and 5 are exclusive of neutral (see Neutral GSL Synthesis in the text) and acidic (see Acidic GSL Synthesis in the text) GSLs, respectively.

Glycosphingolipids (GSLs) are key components of the plasma membrane and are involved in cellular processes crucial for fungi, such as growth, differentiation, signal transduction, and pathogenesis (15). The basic structure of these compounds consists of a sphingoid base backbone (also called long-chain base [LCB], highlighted in blue in Fig. 1A) linked to a fatty acid chain (highlighted in black in Fig. 1A) through an amide bond, forming ceramide, which is then linked through a glycosidic bond to a polar head group, represented by one or more sugar units (highlighted in red in Fig. 1A) (16). In the last few decades, GSLs have been isolated from distinct fungal species, such as A. fumigatus, P. brasiliensis, H. capsulatum, and S. schenckii. Two major classes of GSLs were identified in these opportunistic pathogens, neutral GSLs (or monohexosylceramides) and acidic GSLs (reviewed in references 17 and 18).

Neutral GSLs are conserved among fungi, plants, and mammals, although structural divergence is found among different species (19). In fungi, neutral GSLs (Fig. 1A) exhibit Δ4-Δ8-9-methyl-sphingadienine as the sphingoid base, usually attached to *N*-2′-hydroxyoctadecanoate or to the unsaturated counterpart *N*-2′-hydroxy-(*E*)-3′-octadecenoate as the fatty acid, and a glucose or galactose residue, to form glucosylceramide (GlcCer) and galactosylceramide (GalCer) (17). Whereas in mammalian cells GlcCer and GalCer are then used to make hundreds of complex sphingolipids, such as cerebrosides, gangliosides, and globosides, in fungi, GlcCer and GalCer are considered the final step of the pathway. In contrast, acidic GSLs include inositol phosphorylceramides (IPCs), which are then used as building blocks for more complex molecules, such as mannosylinositol phosphorylceramide (MIPC) and mannosyldiinositol phosphorylceramide [M(IP)_2_C] (Fig. 1B) and others. The ceramide moiety of the IPCs is usually formed by a 4-hydroxysphinganine (phytosphingosine) as the LCB, attached to a very long fatty acid (VLFA) chain (C_18–26_). The major differences between the ceramide moieties of neutral and acidic GSLs are highlighted in orange and purple in Fig. 1B. The LCBs from acidic GSLs contain a hydroxyl group at C-4 and lack the C_9_-methylation and Δ4- and Δ8-unsaturation present in the LCBs from neutral GSLs (Fig. 1A). Additionally, the distinct classes of GSLs exhibit fatty acid chains of various lengths. The resulting phytoceramide is linked via an inositol-phosphate group to one of the sugar cores of fungal GIPCs: GlcNα1-2Ins, Manα1-6Ins, and Manα1-2Ins. Thus, the acidic GSLs in fungi are structurally diverse and more complex than the neutral GSLs.

More than mere constituents of the plasma membrane, glycosphingolipids are clustered along with sterols in specialized membrane microdomains termed lipid rafts, which play a crucial role in the establishment of the fungal cell polarity (20). Polarized growth is a hallmark of filamentous fungal morphology, as fungal hyphae grow by apical extension, in which vesicles containing precursors required for cell wall growth are transported to the hyphal tip through a network of microtubules and the actin cytoskeleton (reviewed in reference 21). In fact, in Aspergillus nidulans, lipid rafts are observed in hyphal tips (22), and sphingolipid synthesis and localization in these active growth sites seem to be relevant for its differentiation, as the disruption of sphingolipid production by myriocin treatment impairs the establishment of the cell polarity axis in spores and prevents normal hyphal branching in germlings (23). Although the role of sphingolipids in dimorphic fungal biology (e.g., hypha formation and hypha-to-yeast transition at 25°C and 37°C, respectively) remains largely unknown, membrane microdomains enriched with sphingolipids have been isolated from H. capsulatum and P. brasiliensis (17, 24), suggesting that they are involved in signal transduction and yeast-host cell interaction.

## THE SHARED PATHWAY OF GSL SYNTHESIS

GSL synthesis is conserved among fungal species and results from the catalytic action of membrane-bound enzymes located in the endoplasmic reticulum (ER) (25, 26). This section focuses on the first two reactions of GSL synthesis (Fig. 1C, reactions 3.1 and 3.2), which are common to the production of neutral (Fig. 1C, reactions 4.1 to 4.6) and acidic (Fig. 1C, reactions 5.1 to 5.4) GSLs. The first and rate-limiting step is the condensation of palmitoyl coenzyme A (palmitoyl-CoA) and serine, catalyzed by the enzyme serine palmitoyltransferase, producing the intermediate 3-keto dihydrosphingosine (3-keto DhSph) (16). The generation of 3-keto dihydrosphingosine is followed by its reduction to dihydrosphingosine (DhSph) through the activity of the 3-keto dihydrosphingosine reductase. As reported for other fungal species (27–29), the generation of 3-keto dihydrosphingosine seems to be essential for filamentous fungal growth. Although the role of 3-keto DhSph reductase in filamentous and dimorphic biology remains poorly investigated, the deletion of the Candida albicans 3-keto DhSph reductase-encoding gene compromised filamentation (30), suggesting that dihydrosphingosine synthesis may be important for polarized growth.

### 3-Keto dihydrosphingosine synthesis.

In A. nidulans, the gene encoding serine palmitoyltransferase was identified as *lcbA*, named due to its homolog LCB1 in Saccharomyces cerevisiae. The contribution of sphingolipid synthesis to fungal biology was investigated through the generation of a conditional mutant (23). In this strain, *lcbA* is under the control of the promoter of the alcohol dehydrogenase I gene (*alcA*), which is strongly induced when alcohol is the sole carbon source and repressed when glucose is the main carbon source (31). The *alcA*::*lcbA* conidia were not able to grow in glucose-containing medium (*alcA*-repressing conditions), suggesting that sphingolipid synthesis is essential for A. nidulans cell polarity and growth (23).

### Dihydrosphingosine synthesis.

Dihydrosphingosine synthesis has been poorly studied thus far in dimorphic and filamentous fungi. In A. fumigatus, 3-keto DhSph reductase is encoded by the *ksrA* gene (30), but the role of *ksrA* for *Aspergillus* biology remains to be elucidated. The production of DhSph constitutes a branching point in the sphingolipid synthesis, as this compound can generate two distinct pools of ceramide (dihydroceramide and phytoceramide), which are used for the formation of neutral (GlcCer and GalCer) or acidic (IPC and MIPC) glycosphingolipids, respectively. These results strongly indicate that 3-keto DhSph and DhSph synthesis contribute to the fungal viability and filamentation process.

## NEUTRAL GSL SYNTHESIS

To form GlcCer or GalCer, dihydrosphingosine is first N acylated with C_16_ or C_18_, saturated or (*E*)-Δ^3^-unsaturated fatty acids through the catalytic activity of ceramide synthase (Fig. 1C, reaction 4.1), resulting in the production of dihydroceramide. Two ceramide synthases, BarA and LagA, have been described in A. nidulans to play an important role in growth (32, 33). The ceramide pool involved in the neutral sphingolipid synthesis (dihydroceramide) is generated by BarA, identified in a screening of mutant strains resistant to the antifungal polyketide heat-stable antifungal factor (HSAF) (32). The deletion of *barA* reduces growth and causes extensive apical branching, along with a mislocalization of lipid rafts from the hyphal tips (32). The *barA* mutant produces normal amounts of IPCs but totally lacks GlcCer, indicating that neutral GSLs, and not acidic IPCs, contribute to the organization and growth at the hyphal tip, at least in this fungus.

Recently, the BarA homolog Cer1 has been characterized in Cryptococcus neoformans. Interestingly, similarly to the A. nidulans *barA* mutant, the C. neoformans *cer1* mutant also produces normal levels of IPCs but totally lacks neutral GSLs, such as GlcCer (34). The C. neoformans *cer1* mutant was found to be avirulent as it cannot survive in the lung environment, from which it is eliminated within a few days after inhalation. This phenotype was ascribed to the lack of GlcCer and impairment of Pma1 activity, necessary for fungal survival in the host neutral/alkaline and acidic environments, respectively (34). These studies indicate that the GlcCer pathway may be more important than the IPC pathway in the regulation of fungal virulence, even though IPCs are essential for fungal growth (35). In fact, IPCs alone are not sufficient to produce a pathogenic strain in C. neoformans and to promote growth at the hyphal tip in A. nidulans. These studies clearly suggest that BarA and Cer1 represent excellent target candidates for the research and development of new antifungal compounds, which will have a broad spectrum of activity as this ceramide synthase is highly conserved in many fungi.

After dihydroceramide synthesis, a hydroxyl group is inserted at C_2_ of the fatty acid chain, generating OH-dihydroceramide. It is of note that fungal OH-dihydroceramide can be composed of fatty acid chains of distinct lengths and levels of saturation. An interesting feature is the unsaturation at C_3_, which has been reported in neutral GSLs from A. oryzae (36), A. fumigatus (37, 38), Fusarium solani (39), P. brasiliensis (40), H. capsulatum (41), and S. schenckii (42), a modification unique to fungal sphingolipids. The ratio of saturated and (*E*)-Δ^3^-unsaturated 2-hydroxy fatty acid can vary among the GSLs from different fungal morphotypes. In fact, only 15% of the total GlcCer extracted from the P. brasiliensis yeasts is composed of (*E*)-Δ^3^-unsaturated fatty acids, while 50% of the total GlcCer contains the Δ^3^-unsaturation in P. brasiliensis mycelium (37). Similarly, a higher proportion of saturated fatty acids was described in the yeast GlcCer from H. capsulatum (41), and the GlcCer from H. capsulatum mycelium is almost exclusively constituted by the (*E*)-Δ^3^-unsaturated 2-hydroxy fatty acids (41). The higher content of (*E*)-Δ^3^-unsaturated GlcCer in mycelial forms of P. brasiliensis and H. capsulatum may be ascribed to the activation of desaturase activity that has been observed during the yeast-to-hypha transition (41), suggesting that (*E*)-Δ^3^-unsaturation of the fatty acid may be involved in signaling pathways that control morphological switch.

The saturation of the sphingosine backbone is also important. Previous studies in C. neoformans showed that a mutant making only saturated GlcCer (*sld8*) is more susceptible to membrane stressors and has increased membrane permeability, even though biophysical studies showed that saturated GlcCer produced more lipid rafts than unsaturated GlcCer species (43). These studies clearly suggest a connection between GSL saturation and fungal biology and pathogenesis, although the molecular mechanisms regulating these effects await further characterization.

### OH-ceramide synthesis, LCB Δ8-unsaturation, and C_9_-methylation.

The next step of the neutral sphingolipid synthesis consists of the C-4 reduction in the sphingoid base of OH-dihydroceramide by the enzyme sphingolipid Δ4-desaturase (Fig. 1C, reaction 4.2), which occurs in the cytosolic face of the ER and generates OH-ceramide (44–46). Then, a double bond between C_8_-C_9_ and a methyl group at C_9_ are introduced in the LCB by sphingolipid Δ8-desaturase (Fig. 1C, reaction 4.3) and sphingolipid C_9_-methyltransferase (Fig. 1C, reaction 4.4), respectively, forming OH-Δ8-9-methyl-ceramide (47–49). The Δ8-unsaturated and C_9_-methylated sphingoid base is characteristic of fungal GlcCer and GalCer, distinguishing them from the mammalian counterparts, which exhibit sphingosine as the LCB (50). These structural modifications found in the fungal LCB are required for normal growth, differentiation, and pathogenesis. The disruption of the A. nidulans Δ8-desaturase-encoding gene (*sdeA*) leads to an accumulation of saturated (and unmethylated) GlcCer, reduced growth, and attenuated virulence in Galleria mellonella larvae (51), similarly to what is observed in C. neoformans (43). The phylogenetic profiling of genes encoding fungal C_9_-methyltransferases in filamentous fungi revealed the presence of two genes in A. nidulans (*smtA* and *smtB*) and Fusarium graminearum (Fg*MT1* and Fg*MT2*), while just one candidate was identified in Neurospora crassa (48). Surprisingly, the deletion of F. graminearum Fg*MT1* neither compromises the synthesis of C_9_-methylated GlcCer nor impairs mycelial growth (52). In contrast, the ΔFg*mt2* mutant produces 65 to 75% of unmethylated GlcCer and 25 to 35% of methylated GlcCer, showing severe growth defects compared to the wild-type strain (52). These results suggest that F. graminearum Fg*MT2* encodes a predominant C_9_-methyltransferase.

While high levels of unmethylated GlcCer are observed in both A. nidulans Δ*smtA* and Δ*smtB* mutants, only the deletion of *smtB* is followed by a reduction in 50% of C_9_-methylated GlcCer content (51), therefore corroborating the existence of a predominant C_9_-methyltransferase in filamentous fungi. Additionally, the *smtA* deletion combined with the *smtB* conditional repression remarkably compromises filamentous growth (51). In agreement with the observation for F. graminearum, this result suggests that C_9_-methyltransferases are essential for filamentous fungal growth/differentiation. Pathogenic yeasts contain only one C_9_-methyltransferase, and its deletion results in a mutant with attenuated virulence (53, 54). Interestingly, certain plant defensins require the C_9_-methylation for fungal GSL recognition (51, 55). Because C_9_-methylation is fungus specific, plant defensins may have a therapeutic potential for treatment of fungal infections.

### GlcCer and GalCer synthesis.

The last step of the pathway involves the transfer of a sugar residue in the Golgi apparatus from UDP-glucose or UDP-galactose to the ceramide backbone by glucosylceramide synthase (Fig. 1C, reaction 4.5) or ceramide galactosyltransferase (Fig. 1C, reaction 4.6), respectively (19, 56). Therefore, the final products contain 9-methyl-4,8-sphingadienine as the sphingoid base attached to 2′-hydroxyoctadecanoic or 2′-hydroxy-3-octadecenoate fatty acid and glucose or galactose as a polar head group. The structural characterization of GSLs from fungi revealed the presence of GlcCer in A. nidulans (57), F. solani (39), F. graminearum (58), H. capsulatum (41), P. brasiliensis (37), and B. dermatitidis (50), while the occurrence of both GlcCer and GalCer has been reported in A. fumigatus (37, 38), A. oryzae (59), and S. schenckii (42) so far. Although GlcCer has been isolated from several fungal species, the role of the glucosylceramide synthase (GCS) in polarized growth has been investigated only in the filamentous A. fumigatus, A. nidulans, and F. graminearum. Its function remains unexplored in dimorphic fungi.

**FIG 2:**
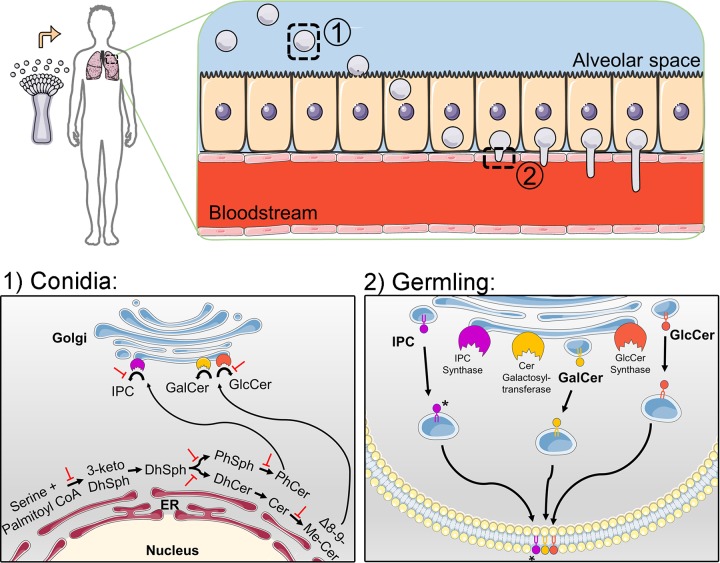
Role of glycosphingolipids in *Aspergillus* biology and pathogenesis. The filamentous fungus *Aspergillus* is ubiquitously dispersed in the environment, and the asexual sporulation produces airborne conidia. The inhalation of *Aspergillus* conidia (1) by a severely immunocompromised host leads to germination and extensive hyphal growth in the lungs (2), which can culminate in disseminated infection through the bloodstream. In growing conidia (1), PhCer and Δ8-9-Me-Cer are produced in the endoplasmic reticulum (ER) and transported to the Golgi apparatus for synthesis of GSLs, such as GalCer, GlcCer, and IPC. The inhibition of steps of this pathway, by gene deletion or antifungal drugs, prevents germination or leads to loss of viability. In the absence of inhibition, IPC, GlcCer, and GalCer can be directed to the active sites of the plasma membrane, playing a role in polarized growth and contributing to fungal invasion (2). The asterisk indicates that after synthesis, IPC can achieve further complexity through the addition of glucosamine, mannose, and galactofuranose residues before being anchored in the plasma membrane.

In filamentous fungi, the pharmacological inhibition of glucosylceramide synthase enzyme by 20 µM d-threo-3P,4P-ethylenedioxy-P4 (EDO-P4) compound prevents the germ tube emergence in A. fumigatus and impairs the hyphal extension in A. nidulans germlings (57). Furthermore, the deletion of the A. nidulans gene encoding the glucosylceramide synthase (*gcsA*) abolishes GlcCer production and reduces filamentation (51). Similarly, the disruption of the F. graminearum FgGCS1 gene was followed by the lack of GlcCer production and compromised growth (58), suggesting that glucosylceramide synthase expression is crucial for the establishment and maintenance of the polarity axis in filamentous fungi (Fig. 2).

In contrast to the GlcCer synthases, whose sequences were previously identified in a wide range of species (56), the gene encoding ceramide galactosyltransferase has been cloned only from rat brains (60), and its fungal homolog, as well as its biological function, remains unknown. Interestingly, the production of GalCer can vary among the dimorphic morphotypes: while S. schenckii mycelia synthesize only GlcCer, both GlcCer and GalCer are found in the lipid extract of the yeast forms (42), suggesting that the ceramide galactosyltransferase may be activated during the S. schenckii mycelium-yeast switch or inactivated during the yeast-to-hypha transition. It has been previously shown that sphingolipids regulate the activity of protein kinases, such as protein kinase C (PKC), and protein phosphatases, involved in signaling cascades that ultimately modulate cell growth, differentiation, and proliferation (61). Similarly, the differential (*E*)-Δ^3^-unsaturation of fatty acids and GalCer production may constitute a mechanism of GSL control over fungal morphogenesis through the activation/deactivation of signal transduction pathways.

After the synthesis in the Golgi apparatus, GSLs are transported to the their final location, which for the most part is the plasma membrane (reviewed in reference 62). In addition, GlcCer can also be found in the fungal cell wall (63) and in vesicles that are exported to the extracellular environment (extracellular vesicles [EVs]) (64). These vesicular compartments were first reported in C. neoformans (65) and, since then, have been identified in many fungi, including the dimorphic species P. brasiliensis (66) and S. schenckii and H. capsulatum (67). The lipid and proteomic analyses of fungal EVs revealed the presence of several virulence factors, such as sterols and proteins involved in pathogenesis and the immune response (64). Although the secretion of EVs seems to be a conserved mechanism across fungal species, their presence in filamentous fungi remains to be elucidated.

## ACIDIC GSL SYNTHESIS

The production of acidic GSLs in fungi is structurally more diverse and more difficult to analyze as standards to be used by mass spectrometry are not commercially available. The final products of this arm of the GSL pathway are IPCs, GIPCs, and MIPCs (and perhaps more), and they have been involved in dimorphic and filamentous fungal biology and virulence.

### Phytosphingosine synthesis.

The synthesis of acidic GSLs starts with the hydroxylation of the DhSph sphingoid base on C-4, catalyzed by the enzyme sphingolipid C_4_ hydroxylase and which generates phytosphingosine (PhSph) (Fig. 1C, reaction 5.1). PhSph production seems to be crucial for filamentous fungal growth. In A. nidulans, sphingolipid C_4_ hydroxylase is encoded by the *basA* gene and the *basA1* mutant was identified in a chemical genetic screening showing hypersensitivity to the antifungal polyketide HSAF (32). In the absence of HSAF, the *basA1* mutation impairs fungal growth at 42°C, which is partially restored by the addition of exogenous PhSph to the culture medium or by the complementation of the mutant strain with the wild-type allele (68). Moreover, the repression of the *basA* gene in the *alcA*::*basA* conditional mutant abolishes fungal growth (68). These results suggest that PhSph synthesis is essential for *Aspergillus* viability and, therefore, may indicate that filamentous fungi are unable to use DhSph in the synthesis of complex GSLs.

Echinocandins constitute a class of antifungal drugs, including caspofungin and micafungin, which interact with hot spots of the integral protein β-1,3-glucan synthase (Fks) and inhibit its activity, compromising cell wall synthesis (69). Very interestingly, the A. nidulans *basA1* mutant exhibits a caspofungin reduced susceptibility (CRS) but a micafungin increased susceptibility (MIS), and this phenotype is reversed by adding myriocin, an inhibitor of serine-palmitoyl transferase, to the cell suspension (70). In addition, previous observations in clinical isolates of *Candida glabrata* demonstrated that CRS-MIS is associated with higher levels of DhSph and PhSph (71). Together, these results suggest that the CRS-MIS phenotype observed in the A. nidulans *basA1* strain can be attributed to the accumulation of DhSph and indicate that sphingolipids may interact with echinocandins or/and with Fks. They also suggest that the effect of echinocandins on sphingolipids seem to be restricted to IPCs, as C. glabrata does not make any GlcCer (72).

### Phytoceramide and IPC synthesis.

Next, a very long fatty acid (VLFA) chain containing 18, 24, or 26 carbons is amide linked to phytosphingosine by the ceramide synthase, forming phytoceramide (PhCer) (Fig. 1C, reaction 5.2) (49, 73, 74). PhCer production seems to be relevant for fungal viability and hyphal morphogenesis. In A. nidulans, the ceramide synthase that generates the phytoceramide pool is encoded by the *lagA* gene (32), and the contribution of LagA to the fungal growth was assessed in a conditional *alcA*::*lagA* mutant. Under *lagA*-repressing conditions, the *alcA*::*lagA* strain shows a striking reduction in growth and distorted hypha, suggesting that *lagA* is an essential gene and controls the polarized growth (32).

Phytoceramide is then used as the substrate for the synthesis of complex sphingolipids, which occur in the Golgi apparatus. PhCer is transported from the ER to the outer leaflet of the Golgi membrane (as illustrated in Fig. 2), by both vesicle-dependent and independent mechanisms, and is then flipped to the Golgi inner membrane (75). The first reaction involves the transference of the *myo*-inositol-1-phosphate group from the phosphatidylinositol to the C_1_ hydroxyl of phytoceramide, generating IPC (Fig. 1C, reaction 5.3). The inositol-phosphate attachment is catalyzed by the IPC synthase (Fig. 1C, reaction 5.3), encoded by the *aurA* gene (from aureobasidin A resistance) in *Aspergillus* species (76), also called *IPC1*, which is essential in fungi. The production of IPC is crucial for fungal viability. Repression of *aurA* in A. nidulans *alcA*::*aurA* spores prevents germination, causing a terminal phenotype (23).

The integrity of the IPC synthesis pathway is relevant for filamentous fungal viability, not only due to the role of IPC in fungal differentiation but also because the level of DhSph, PhSph, and PhCer needs to be highly regulated, as DhSph and PhSph are highly toxic to fungal cells. In addition, these molecules act as signaling molecules in distinct processes such as heat stress adaptation, endocytosis, and apoptosis (77, 78). Thus, changes in their level may have uncontrolled effects on signaling events, resulting in fungal cell death. Indeed, DhSph and PhSph possess robust antifungal activity against A. nidulans, and the addition of these sphingoid bases to the culture medium induces DNA condensation, DNA fragmentation, and phosphatidylserine externalization, characteristic hallmarks of apoptotic cell death (79). In Neurospora crassa, phytoceramides containing C_18_ and C_24_ fatty acid chains are produced in response to the combined stresses of heat shock and 2-deoxyglucose treatment, suggesting that ceramides mediate the signaling of fungal cell death (80).

These observations indicate that PhCer synthase and IPC synthase constitute potential targets for the development of new antifungal drugs, as inhibiting these enzymes will lead to the accumulation of PhSph and PhCer and, ultimately, fungal death. In fact, the inhibition of A. nidulans IPC synthase by aureobasidin A is followed by an accumulation of ceramide and cell cycle arrest (23). Aureobasidin A also showed antifungal activity against H. capsulatum, B. dermatitidis, A. nidulans, and Aspergillus terreus (81), in addition to Candida albicans and Cryptococcus neoformans (81, 82). Because mammalian cells lack Ipc1, this enzyme represents an ideal antifungal target (83).

### GIPC synthesis.

Further IPC processing by glycosyltransferases generates glycosylinositol phosphorylceramides (GIPCs), anionic glycosphingolipids which are found in several fungi and are particularly regulated during morphogenesis (17, 18, 84). The glycan moieties of the fungal GIPCs show great diversity and complexity, varying among species and dimorphic morphotypes. Nonetheless, glucosamine and mannosyl residues are commonly linked to the inositol group of the IPC, forming three carbohydrate “cores” used as building blocks for GIPCs: (i) the glucosamine-α-1,2-IPC (GlcNα2IPC), (ii) mannose-α-1,6-IPC (Manα6IPC), and (iii) mannose-α-1,2-IPC (Manα2IPC) (17). To date, the simultaneous expression of these three core linkages has been reported only in S. schenckii GIPCs, although in distinct fungal morphotypes (85, 86). While the GIPCs isolated from the S. schenckii mycelium are constituted by the Manα6IPC and Manα2IPC cores, the most abundant GIPC from the yeast form is composed of the GlcNα2IPC core, indicating that the differential expression of the GIPCs may contribute to the dimorphic transition (85, 86). The zwitterionic GlcNα2IPC core of GIPCs has also been described in A. fumigatus, which, as in S. schenckii, is elongated by the addition of two mannose residues to produce Manα1,3Manα1,6GlcNα1,2IPC (87, 88). In A. fumigatus, the glucosamine head group is attached to the IPC molecule through the activity of the *N*-acetylglucosaminyltransferase (UDP-GlcNAc:IPCα1,2GlcNAcT) GntA, and the deletion of the *gntA* gene was found to abolish Manα1,3Manα1,6GlcNα1,2IPC synthesis (89).

Although previously observed in mycobacteria (90), in fungi GIPCs possessing the α-1,6-linked mannose core seem to be exclusive of S. schenckii (86, 91). In contrast, the mannose-α-1,2-IPC structure has been isolated from S. schenckii (86), P. brasiliensis (92), H. capsulatum (93, 94), A. fumigatus (87, 88), and A. nidulans (95). The α-1,2-mannosylation of the IPC backbone is promoted by the enzyme GDP-mannose:inositol-phosphorylceramide transferase, generating mannosyl inositol phosphorylceramide (MIPC). In A. fumigatus, the deletion of the gene encoding the MIPC transferase (*mitA*) abolishes the production of MIPCs and leads to the accumulation of IPC (96). Surprisingly, the Δ*mitA* mutant exhibits radial growth and virulence comparable to the wild-type strain, suggesting that MIPC is not critical for fungal differentiation and pathogenesis (96). Further structural complexity can be achieved through the addition of mannose, galactofuranose (Gal*f*), and choline-phosphate groups to the MIPC molecule. In fact, a compound (Manα1,3Gal*f-*β1,6Manα1,2IPC) containing the Gal*f*β1,6Manα1,2IPC structure has been identified in both dimorphic forms of P. brasiliensis and named Pb-1 (92). Interestingly, a high titer of anti-Pb-1 antibodies has been detected in the sera of patients with paracoccidioidomycosis, and the Pb-1 reactivity is reduced after the oxidation of the Gal*f* residue (97). Two A. fumigatus GIPCs exhibiting the Gal*f*-β1,6Manα1,2IPC motif have also been isolated, including Af-3b, which is structurally identical to P. brasiliensis Pb-1 and has been proposed to be the synthetic precursor of Af-4 (Manα1,2Manα1,3Gal*f*-β1,6Manα1,2IPC) (87). Like Pb-1, Af-3b and Af-4 are also recognized by MEST-1, a mouse monoclonal antibody which binds to Gal*f-*β residues (87, 98). Although the specific enzymes involved in the synthesis of these complex sphingolipids await further identification and characterization, the structural identification of these specific fungal glycosphingolipids may open a new road for the development of specific monoclonal antibodies which can be used for diagnostic or/and therapeutic approaches.

## CONCLUDING REMARKS

This minireview highlights the relevance of GSL synthesis to fungal growth and pathogenesis and suggests that the enzymes of this pathway may represent promising targets for the development of new antifungal drugs.

In filamentous fungi, impairment of GlcCer synthesis reduces growth, differentiation, and virulence. Therefore, small molecules capable of inhibiting GlcCer synthase constitute promising candidates for antifungal therapy. In fact, acylhydrazones inhibiting the synthesis of fungal but not mammalian GlcCer have been recently described as potent antifungal agents with a broad spectrum of activity and low toxicity to mammalian cells (99, 100). Interestingly, these compounds were found to be efficacious against invasive cryptococcosis, candidiasis, and aspergillosis in a murine model of infection (99, 100). Another important aspect of neutral GSLs is the fatty acid unsaturation and the sphingosine unsaturation and C_9_-methylation of LCB. Targeting the enzymes involved in these processes, which are also fungus specific, may impair fungal growth in the host and, ultimately, improve the outcome of the infection.

The production of IPC is also crucial for filamentous growth, and the inhibition of PhCer and IPC synthesis leads to the accumulation of PhSph and PhCer intermediates, inducing fungal apoptosis. Therefore, the design of new compounds that target these enzymes constitutes a promising alternative in antifungal therapy to be pursued.

Although the role of the GSL pathway in dimorphic fungi remains largely understudied, GSLs are clearly involved in the regulation of the yeast-to-hypha transition in this class of fungi. Structural differences between fungal and mammalian GSLs exist, and as we improve our method of detection, their structural features could be exploited for the isolation of specific plant defensins or/and for the generation of specific monoclonal antibodies to be used as new therapeutic strategies.
